# Vaginal Ring Pessary Migration and Embedment With Rectal Prolapse: A Rare Complication of a Forgotten Ring Pessary in an Elderly Patient

**DOI:** 10.7759/cureus.85187

**Published:** 2025-06-01

**Authors:** Yi Jie Chew, Hui Men Selina Chin, Shao Nan Khor, Shau Khng Jason Lim, Wai Yen Lee

**Affiliations:** 1 Department of Obstetrics and Gynaecology, Singapore General Hospital, Singapore, SGP; 2 Department of Colorectal Surgery, Singapore General Hospital, Singapore, SGP

**Keywords:** procidentia, rectal prolapse, rectovaginal fistula, retained ring pessary, uterine prolapse

## Abstract

The use of a vaginal ring pessary is a common nonsurgical option for the management of pelvic organ prolapse. It may sometimes be considered a first-line option for patients who are not suitable surgical candidates. Pessaries generally have a good safety profile, and serious adverse complications are uncommon. However, long-term use without appropriate follow-up may lead to rare and severe complications. We report a case of vaginal ring pessary migration and embedment with rectal prolapse in an 81-year-old woman. She presented to the Emergency Department complaining of a lump in the perineum. She had a background of pelvic organ prolapse, for which a ring pessary was inserted more than 16 years ago. On examination, she was found to have procidentia and a grade 4 rectal prolapse, with a vaginal ring pessary embedded through the low rectum. The pessary was removed under general anesthesia, and no patent rectovaginal fistula was observed. She made an uneventful recovery and was discharged on the third day of admission. This rare complication of a retained vaginal pessary underscores the importance of follow-up and the need for a recall system for patients using vaginal pessaries. It also highlights that patient selection is key when offering vaginal pessary as a treatment option for pelvic organ prolapse.

## Introduction

Pelvic organ prolapse is a common condition often seen in postmenopausal and multiparous women. It occurs when the pelvic floor muscles and their surrounding ligamentous supports become weak and can no longer support the pelvic organs adequately [1].

Prolapse of the uterus can occur on its own or may be associated with prolapse of the anterior (cystocele) or posterior (rectocele) vaginal wall. Women who have undergone a hysterectomy may develop prolapse of the vaginal vault [2]. The incidence of pelvic organ prolapse increases with age, from 9.7% in women aged 20-39 years to 49.7% in women aged 80 and older [3]. Rectal prolapse is relatively uncommon, with an incidence of 0.5% [4].

Once prolapse occurs, it is often irreversible. Women with mild symptoms are usually managed conservatively with pelvic floor exercises. Women with more severe prolapse are typically offered treatment options including vaginal pessary or surgical intervention [5]. A vaginal pessary may be preferred for patients who decline surgery or are unfit for surgery due to existing comorbidities.

## Case presentation

We report a case of a community-ambulant 81-year-old woman with one previous normal vaginal delivery who presented to the Emergency Department complaining of a protruding lump in the perineum. She had a past medical history of hypertension, hyperlipidemia, bilateral cataracts, and age-related macular degeneration. Her BMI was 23.6. She denied any past surgical history. The patient reported feeling the lump in the perineum for the past week but did not seek medical attention earlier. The lump appeared spontaneously, and there was no preceding straining to defecate or pass urine. She denied any urinary or fecal incontinence. There was no associated per rectal or per vaginal bleeding. She came to the hospital because the lump was affecting her ability to walk.

The patient was first seen 16 years ago with procidentia: complete prolapse of the uterus beyond the level of the hymen distally. This was associated with urinary urgency and contact bleeding. She was initially planned for surgery, but a ring pessary (size 74 mm) was eventually inserted. She lived alone and was lost to follow-up, not knowing that follow-up was required after the ring pessary insertion. There was no known history of cognitive disorders that could have contributed to her missing the scheduled appointments. There was no history of expulsion or reinsertion since the pessary was inserted.

**Figure 1:**
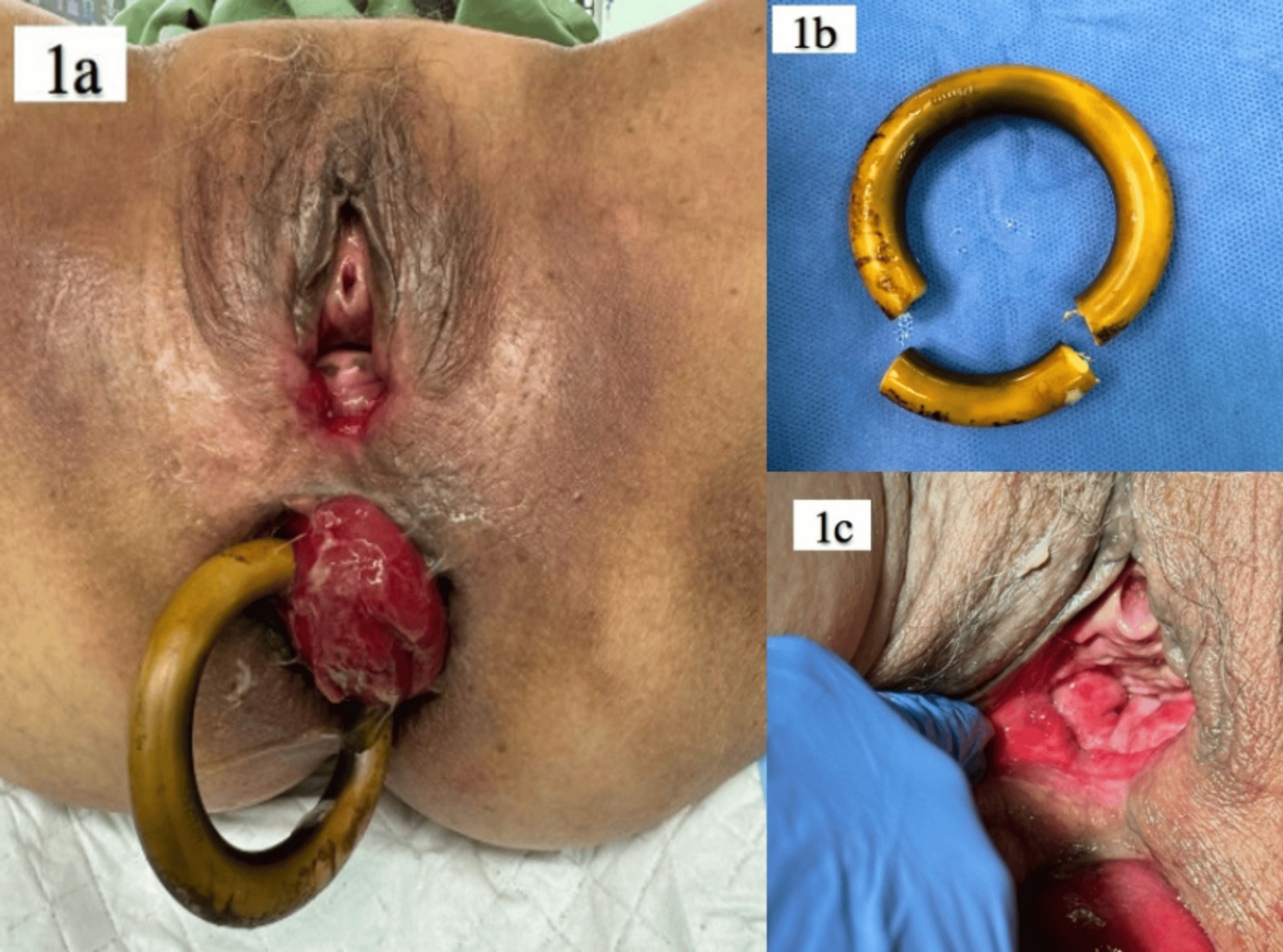
Ring pessary encased within prolapsed rectum (a) and ring pessary that was cut and removed (b). Procidentia was reduced into vagina to show a sealed rectovaginal fistula (c).

A gynecological consult was requested in the Emergency Department. On examination, stage 4 uterovesical prolapse (procidentia) was noted, along with a grade 4 full-thickness rectal prolapse. No ulceration was noted, and the surrounding skin appeared healthy. The procidentia was reducible but recurred spontaneously. A pessary ring was seen embedded within the prolapsed rectal wall (Figure 1). Per vaginal examination revealed a small indentation in the posterior vaginal wall, possibly a sealed rectovaginal fistula (Figure 1c).

**Figure 2:**
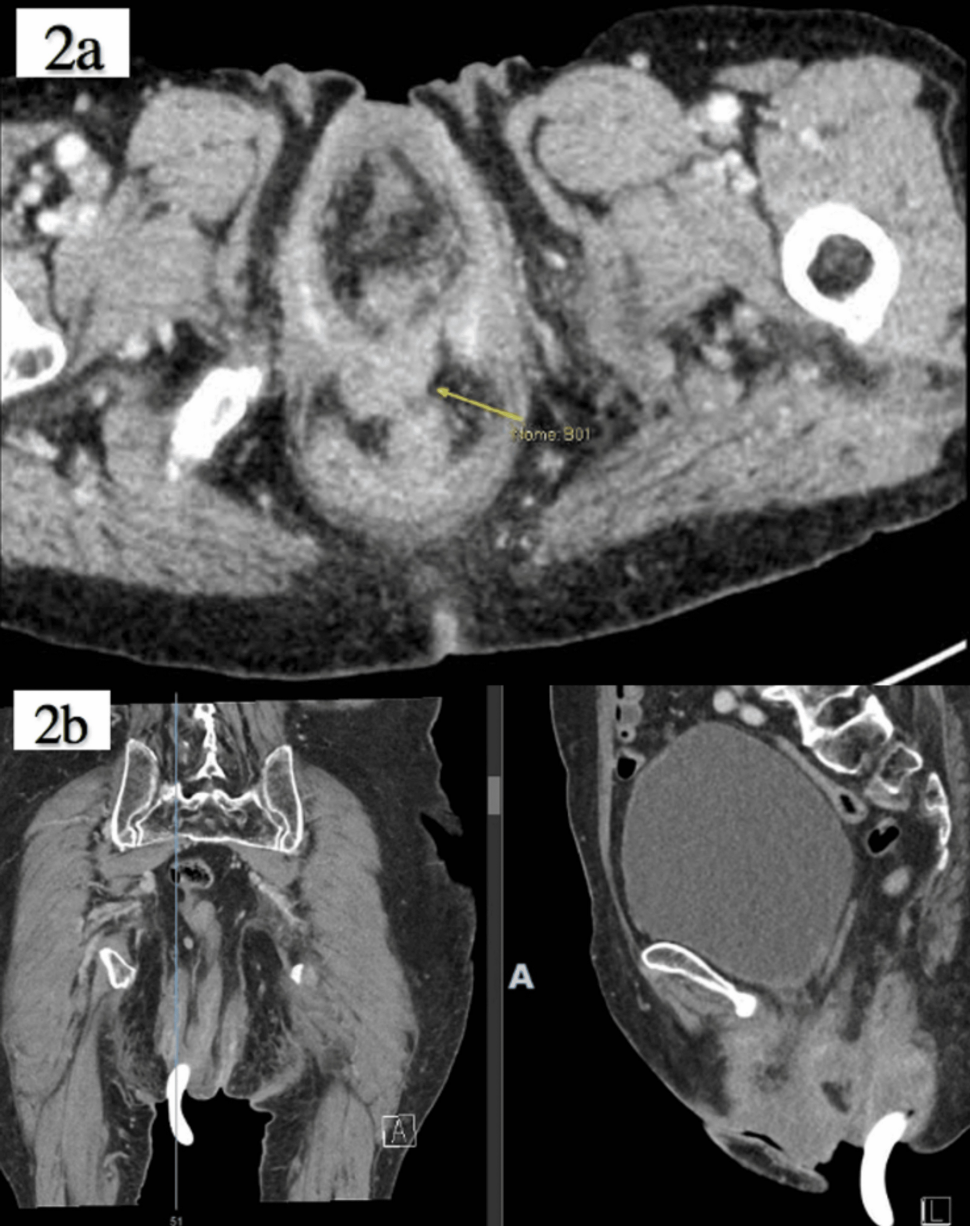
CT scan showing the chronic rectovaginal tract (a) and a pessary ring seen in prolapsed rectum: coronal view and sagittal view (b).

The patient was then referred to Colorectal Surgery. A computed tomography (CT) scan of the abdomen and pelvis revealed that the pessary ring appeared attached to the right wall of the prolapsed anorectum, and there was a focal soft tissue density anterior to the rectum, which may have represented a chronic rectovaginal tract that had been sealed off from the vagina (Figure 2 a, b).

Uncomplicated diverticulosis was noted, but no abdominal or pelvic masses were seen. The patient subsequently underwent examination under general anesthesia and removal of the prolapsed pessary ring the next day. Intraoperatively, the vaginal pessary was seen penetrating the low rectal mucosa without full-thickness rectal wall penetration. The rectal mucosa was prolapsed and incarcerated in the presence of the pessary. The rectal mucosa appeared edematous with congestion, but without frank ischemia. The posterior vaginal wall was thickened and fibrotic, but there was no palpable dimple or defect.

The pessary ring was cut and removed using a bone cutter (Figure 1b). Upon removal of the pessary ring, the rectal prolapse reduced spontaneously. A mucosal defect was seen anteriorly, 4-5 cm proximal to the anal verge. A blinded fibrotic tract with a tunnel measuring 3-4 cm cranially was noted. A 1 cm superficial ulceration was observed on the low rectal mucosa. There was no full-thickness perforation, and only a mucosal defect was identified.

The patient made an uneventful recovery and was discharged three days later. She was reviewed in clinic one month later and reported normal bowel movements. No rectal prolapse was noted. Digital rectal examination did not demonstrate any obvious defect or fistula.

**Figure 3:**
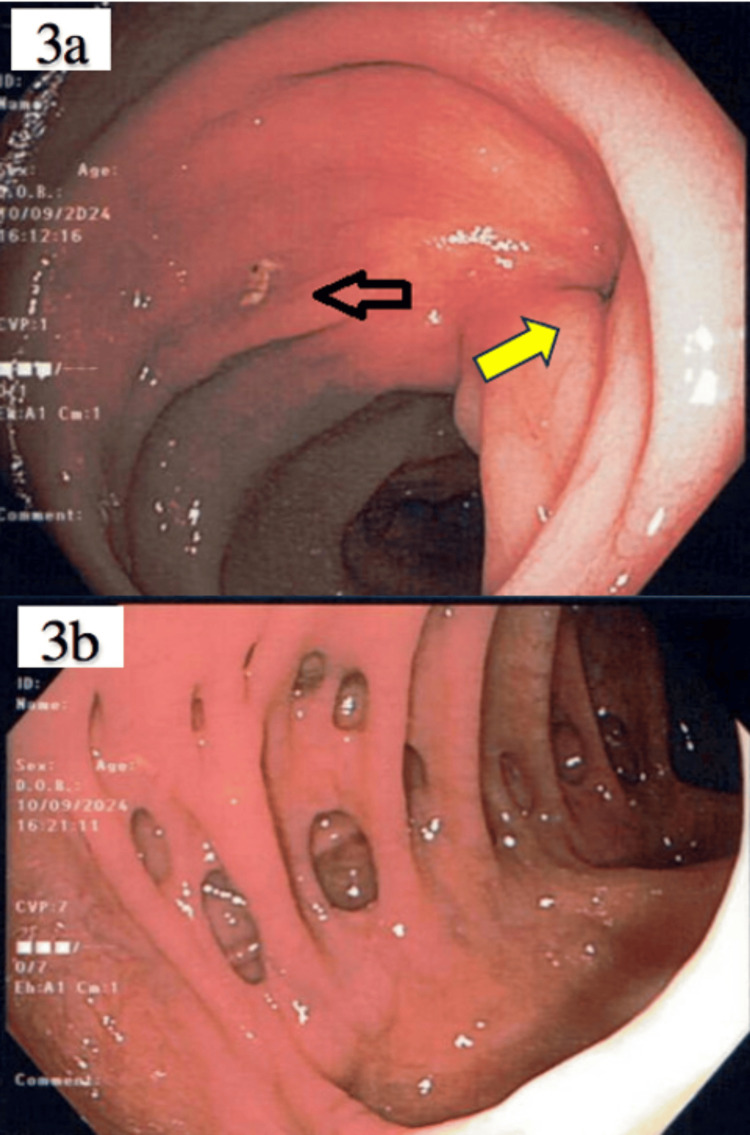
Dimpling over pessary exit site on colonoscopy two months after surgery (a, yellow arrow). Mucosal ulceration is still present but smaller (a, black arrow). Diverticulosis seen on colonoscopy, consistent with CT findings (b).

A follow-up colonoscopy was performed two months after discharge, which showed only rectal mucosal dimpling at the previous pessary exit site and diverticulosis (Figures 3a, 3b).

## Discussion

The prevalence of pelvic organ prolapse in women is up to 40%. The efficacy of vaginal pessaries is reported to be 90.7% [6]. It is a noninvasive alternative for women who wish to avoid surgery or are poor surgical candidates. The ring pessary has a high continuation rate and provides good improvement in both bulge and irritative bladder symptoms [7].

According to NICE (National Institute for Health and Care Excellence) guidelines, patients who are at risk of complications should be offered an appointment in the pessary clinic every six months. Common complications include bleeding, vaginal discharge, and ring expulsion. Pessaries are also recommended to be removed once every six months to prevent serious complications. At our institution, women who opt for ring pessary insertion are usually given regular six-monthly appointments for examination and pessary change. Failure to do so can result in consequences such as ulceration, bleeding, infection, vaginal stenosis from chronic inflammation, and fistula formation [8]. In the case of a retained pessary, long-term compression of the vaginal wall may lead to ischemia and subsequently necrosis. Over time, erosion of the vaginal wall may cause fistula formation, including uterovesical and rectovaginal fistulas.

Rectovaginal fistula is a rare complication of vaginal pessary use. In 2004, Hanavadi et al. reported a case of a shelf pessary eroding into the rectum through the posterior fornix [9]. The patient presented with feculent vaginal discharge. An anterior rectal wall defect was seen on colonoscopy, and a diverting colostomy was eventually performed. In our case, the patient was fitted with a ring pessary. There was no communication between the vagina and rectum. The prolapsed rectum also facilitated the removal process, as the pessary could be easily visualized. At least two other cases of rectovaginal fistula due to vaginal pessary have been reported [10,11], both involving the use of shelf pessaries, in contrast to the ring pessary used in our case.

## Conclusions

Ring pessaries are commonly used for the management of pelvic organ prolapse. It is crucial that patients are properly educated and demonstrate a clear understanding of the need for follow-up and interval pessary changes before insertion is performed. This case highlights the importance of keeping patients with a pessary ring on follow-up and recalling them for appointments in the event they fail to attend. Proper documentation of the ring size, date of insertion, and follow-up appointment date must be completed by the clinician after pessary insertion.

Although not common, forgotten pessaries can erode into the rectum, and management should be multidisciplinary, involving both the gynecology and colorectal teams. Furthermore, it is important to engage various stakeholders and assess the social setup of such patients, as they may be more likely to default on their follow-up appointments. Clinicians must consider the social and functional status of the patient when offering long-term pessary use as a conservative option for the management of pelvic organ prolapse. Definitive management for pelvic organ prolapse should eventually be discussed by the gynecological team at future follow-ups.
